# Cyanobacterial Light-Harvesting Phycobilisomes Uncouple From Photosystem I During Dark-To-Light Transitions

**DOI:** 10.1038/srep14193

**Published:** 2015-09-21

**Authors:** Volha Chukhutsina, Luca Bersanini, Eva-Mari Aro, Herbert van Amerongen

**Affiliations:** 1 Laboratory of Biophysics, Wageningen University, P.O. Box 8128, 6700 ET, Wageningen, The Netherlands; 2 BioSolarCells, P.O. Box 98, 6700 AB Wageningen, The Netherlands; 3 Department of Biochemistry, Molecular Plant Biology, University of Turku, FI-20014 Turku, Finland; 4 MicroSpectroscopy Centre, Wageningen University, 6703 HA Wageningen, The Netherlands

## Abstract

Photosynthetic organisms cope with changes in light quality by balancing the excitation energy flow between photosystems I (PSI) and II (PSII) through a process called state transitions. Energy redistribution has been suggested to be achieved by movement of the light-harvesting phycobilisome between PSI and PSII, or by nanometre scale rearrangements of the recently discovered PBS-PSII-PSI megacomplexes. The alternative ‘spillover’ model, on the other hand, states that energy redistribution is achieved by mutual association/dissociation of PSI and PSII. State transitions have always been studied by changing the redox state of the electron carriers using electron transfer inhibitors, or by applying illumination conditions with different colours. However, the molecular events during natural dark-to-light transitions in cyanobacteria have largely been overlooked and still remain elusive. Here we investigated changes in excitation energy transfer from phycobilisomes to the photosystems upon dark-light transitions, using picosecond fluorescence spectroscopy. It appears that megacomplexes are not involved in these changes, and neither does spillover play a role. Instead, the phycobilisomes partly energetically uncouple from PSI in the light but hardly couple to PSII.

To optimize their light-harvesting capacity, photosynthetic organisms adapt rapidly to changing light conditions and/or metabolic demands by regulating the distribution of absorbed light energy between photosystems I and II (PSI, PSII). Such changes occur for instance during dark-light transitions^1^ but also as a response to changes in light quality, causing the so-called state transitions^2^,^3^,^4^. State transitions can be defined as a change in the relative antenna size of PSI and PSII as a response to a change of the redox status of intersystem electron carriers. State 1 is induced by oxidation of intersystem electron carriers, usually upon excess excitation of PSI as compared to PSII. State 2 is induced by reduction of intersystem electron carriers, either through excess excitation of PSII or by dark respiratory pathways^1^. Supposedly, light-harvesting phycobilisomes (PBSs) can shuttle between both photosystems in order to actively balance PSI and PSII activity, like the light-harvesting complex II in plants^2^,^5^,^6^,^7^.

A close relation between dark-light transitions and state transitions has been proposed by Mullineaux and Allen^1^. Experimentally, low-intensity blue or far-red light is commonly used to induce state 1, while a short period of pre-illumination with low-intensity orange light or of darkness is used to put cells into state 2^8^,^9^. It has been suggested that state 1 can also be induced by any colour of the light after a period of darkness^1^, whereas darkness brings the cells back to state 2^10^,^11^. However, a clear mechanism of dark-light transitions is not demonstrated and so far it is unknown whether both transition phenomena correspond to the same underlying physical process.

Decoupling of PBSs from PSs is another regulation mechanism of cyanobacteria to modulate the excitation energy arriving at the PSs, in particular in different stress conditions (for a review see^12^). It was shown that the exposure of *Synechocystis PCC 6803* (hereafter Synechocystis) cells to strong light leads to energetic uncoupling and detachment of PBSs from the reaction centres, at least from those of PSII^13^,^14^,^15^. A similar effect was also observed in mutants with a destabilized PSII structure^16^,^17^,^18^. In strong light, disruption of excitation energy transfer (EET) within the PBS antennas has also been reported, indicating dissociation of phycobiliproteins from the PBS body^13^.

Olive and co-workers found that in the cyanobacterial thylakoid membrane in state 1, PSII complexes are arranged in row-like superstructures, while in state 2 the organization of PSII is more random^19^. Based on this observation, the “spillover” model of state transitions was proposed, which assumes that in state 2 excess energy absorbed by pigments associated with PSII “spills over” to PSI, because in this state PSI and PSII have moved sufficiently close to each other^20^,^21^. The spillover model therefore implies that PBSs, or their movement, play no role in state transitions. Recent reports suggest that spillover might be involved in high light conditions^22^,^23^.

After the “spillover” model was proposed, Joshua and Mullineaux were the first to correlate the presence of PBS mobility in cyanobacteria with state transitions. They fixed cyanobacteria in either state 1 or 2 by immersing the cells in a buffer of high osmotic strength, which strongly inhibited PBS diffusion^24^. Substantial differences could be observed in steady-state fluorescence emission spectra at 77 K. The suggestion that PBSs detach from PSI and attach to PSII during the transition from state 2 to state 1 was based on enhanced fluorescence emission from PSII in the 680–695 nm spectral region at 77 K, after normalizing the spectra for state 1 and 2 to their PBS-related emission peak at 665 nm or to the PSI peak at 720 nm^24^,^25^. PBS migration between PSs in that study was also supported by fluorescence recovery after photobleaching (FRAP) measurements, which showed that PBSs diffuse rather rapidly in the cells while PSII is immobile on the time scale of the measurements. From these steady-state fluorescence and FRAP observations, a model with physical redistribution of PBSs during state transitions was suggested. The suggested movement of PBSs from one photosystem towards the other was, however, never explicitly demonstrated: the 695-nm emission peak as observed by Joshua and Mullineaux is indeed characteristic for the PSII antenna CP47, but the peak at 685 nm can contain contributions from both PSII and allophycocyanin (APC_680_) in PBSs^26^,^27^. Furthermore, APC_680_ fluorescence dominates the PBS steady-state spectra both in intact cells and in isolated PBSs^27^,^28^, which makes it difficult to distinguish, with steady-state fluorescence measurements only, between the migration of PBSs from PSI to PSII and the simple detachment of PBSs from PSI. To summarize, PBS mobility plays a role during state transitions but the migration of PBSs between the photosystems is still questionable.

It has also been proposed that both the PBS mobility and the spillover effects contribute to state transitions, but the major contribution comes from PBS mobility^29^,^30^. Based on x-ray structures of PSII, PSI, and allophycocyanin and 77 K steady-state state fluorescence measurements, it was hypothesized that the major part of energy redistribution comes from only slight movements of PBSs and that a single PBS might feed energy to PSI and PSII simultaneously^29^. This idea was supported recently by the isolation and characterization of a fully functional cyanobacterial megacomplex (making use of chemical cross-linking), composed of PBS, PSI and PSII^31^. The authors showed that a single PBS feeds excitations to both PSs within the megacomplex^31^. If such megacomplexes would occur *in vivo,* PBSs would not have to move in order to induce a change in excitation flow from PBS to PSI or PSII. In the isolated PBS-PSII-PSI megacomplex, the rate of excitation energy transfer (EET) to PSI was found to be slower than to PSII.

Here, we studied “dark” and “light” states of the cyanobacterium Synechocystis using steady-state and time-resolved fluorescence spectroscopy. First, dark/light transitions were followed by steady-state fluorescence measurements at 77 K in order to confirm the occurrence of two distinct states. Instead of the commonly used normalization to one of the emission peaks, an external fluorescent calibration probe was used and the spectra were normalized to its fluorescence emission. This allowed us to monitor absolute changes in all fluorescence emission bands. We used picosecond fluorescence spectroscopy^32^,^33^ to explicitly address the questions (1) whether phycobilisomes move between the photosystems, (2) whether cyanobacterial megacomplexes occur *in vivo* and (3) whether spillover occurs during dark-light transitions.

## Results and Discussion

### Changes in PBS-related excitation-energy distribution observed by 77K steady-state fluorescence emission

**Figure 1:**
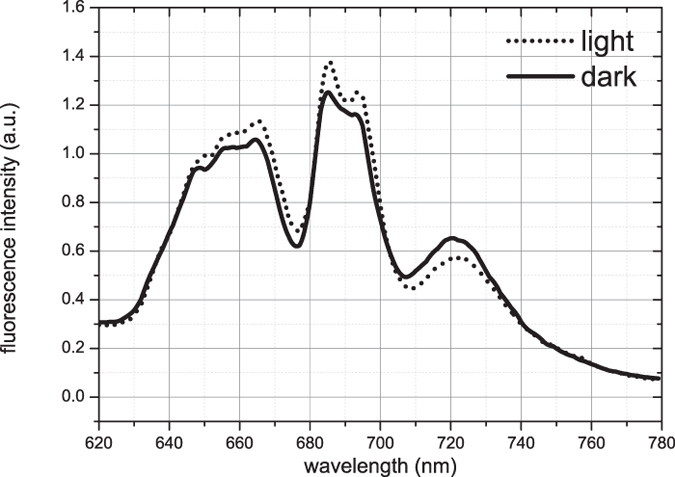
Fluorescence spectra of *Synechocystis sp. PCC 6803* cells measured at 77 K upon 580 nm excitation. The spectra are normalized to the fluorescence emission of the external fluorescent probe Rhodamine B.

First, fluorescence emission spectra of Synechocystis cells were recorded upon PBS excitation (at 580 nm) at 77 K. The spectra of the dark and light state were in both cases normalized to the fluorescence signal of Rhodamine B that was added to the sample for calibration purposes (see Fig. 1). The broad fluorescence emission in the 630–670 nm region originates from phycocyanin (630–665 nm, PC) and allophycocyanin (660–670 nm, APC_660_)^34^ of the PBSs. The maximum at 685 nm corresponds to emission from both the PBS terminal emitter (APC_680_) and PSII^26^,^28^, while the maximum at 695 nm is due to red-shifted Chl *a* emission of CP47 in PSII^35^. The fluorescence emission at 720 nm originates from PSI^31^. Spectra were recorded for cells acclimated to growth light, to darkness and again to growth light conditions, indicating a reversibility of the effects observed in these transitions (Fig S1).

The Synechocystis cells showed pronounced changes upon the light-to-dark transition: in the light state of Synechocystis cells, the fluorescence spectrum clearly shows less PSI emission at 720 nm, but increased emission at 685–695 nm as compared to the dark state. Such differences in the 685–695/720 nm fluorescence ratio are also typically observed when comparing states 1 and 2, where they are thought to indicate increased energy transfer from PBSs to PSII in state 1 and to PSI in state 2 (for example^24^,^36^,^37^). For the light state, an increased fluorescence in the 630–670 nm region was also observed, which must be entirely attributed to PC and APC_660_ in PBSs. Therefore, the spectral differences cannot only be explained by PBS migration from PSI to PSII, if migration lifetimes from PBS to PSs stay the same. To clarify whether PBS energetic uncoupling contributes to the observed spectral changes and/or whether some other processes are involved, we performed time-resolved measurements.

### The two states characterized by time-resolved fluorescence

**Figure 2:**
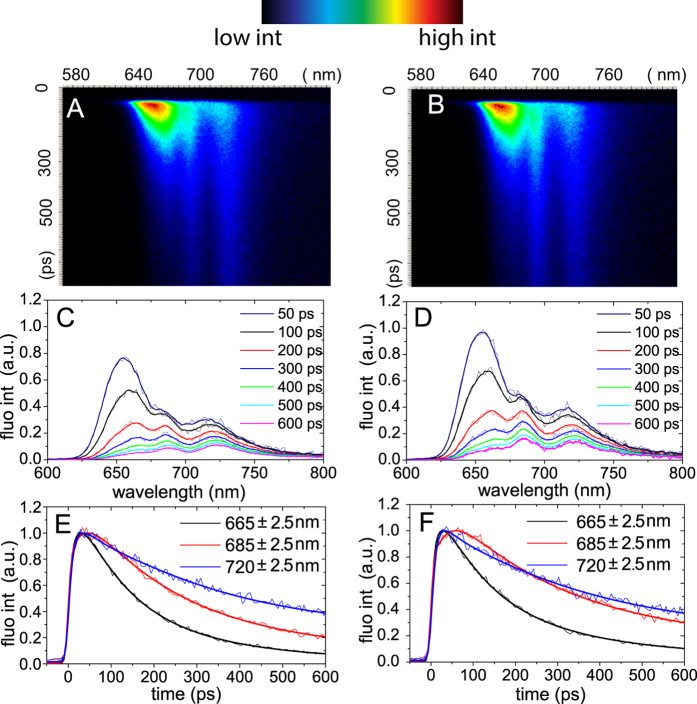
Time-resolved fluorescence of *Synechocystis sp. PCC 6803* measured in dark (A,C,E) and light-adapted states (B,D,F) at 77 K upon 580 nm excitation. (**A**,**B**) Streak-camera images. False colours indicate the fluorescence intensity. (**C**,**D**) Representative fluorescence spectra measured at various delay times after the excitation pulse. (**E**,**F**) Representative decay traces (thin lines) and their fits (thick lines) were taken at three characteristic emission wavelengths: 665 nm (PBS), 685 nm (APC_680_, PSII) and 720 nm (PSI). For better comparison the traces were normalized at their maxima. For description of the used fitting model we refer to Materials and methods.

**Figure 3:**
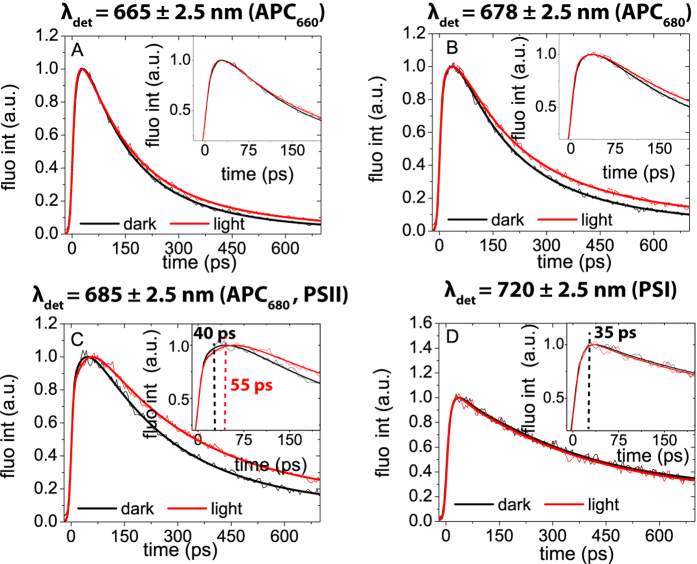
Comparison of original fluorescence kinetic traces (thin lines) and corresponding fitting curves (thick lines) in dark (black line) with those in light (grey line) states, taken at four emission bands: 665 nm (PBS), 678 nm (APC_680_), 685 nm (APC_680_, PSII) and 720 nm (PSI). All measurements were done at 77 K upon 580 nm excitation. The insets zoom in on the first part of the fluorescence kinetics. Each experiment was repeated at least 3 times for different generations of the cell culture. All differences for the two states, measured for different generations, were found to be very reproducible (Fig S2).

Synechocystis cells, locked in light and dark states, were monitored by recording their time-resolved fluorescence emission spectra upon 580 nm excitation at 77 K. The obtained streak-camera images are presented in Fig 2 together with several representative fluorescence decay traces and fluorescence spectra at various delay times. The spectral resolution of time-resolved data is 5 nm (as opposed to 2 nm for the steady-state data). Like in the steady-state fluorescence emission spectra, three distinct emission bands are observed in the streak-camera data with maxima at 665, 685, and 720 nm. Again, these peaks are associated with PC and APC_660_, APC_680_ and PSII, and PSI, respectively. In Fig. 3 the fluorescence at the three emission maxima is compared for the two states. Additionally, the difference at 678 nm was plotted to specifically illustrate the difference of APC_680_-fluorescence (Fig. 3B).

The above-given suggestion that the higher intensity in the 650–670 nm region for the light state (Fig. 1) is due to PBS energetic uncoupling is confirmed by comparison of the fluorescence decay kinetics of two PBS-related emission wavelengths (665 and 678 nm) for the two states (Fig. 2 and Fig. 3A,B). The final parts of the fluorescence traces (0.2–0.7 ns) at 665 nm (PC and APC_660_, Fig. 3A) and at 678 nm (APC_680_, Fig. 3B) in the light state decay slower than in the dark state, which indicates a higher contribution of long-living components in the light state, corresponding to uncoupled antenna. This effect leads to the observed increase of steady-state fluorescence in the PC (630–655 nm) and APC (660–670 nm, ~685 nm) wavelength regions.

Subsequently, we looked at the differences between the fluorescence kinetics in the light and dark states, measured at PS-related wavelengths (PSII: 685 nm (Fig. 3C), PSI: 720 nm (Fig. 3D)). We found that both for the dark and the light state the PSI fluorescence reaches its maximum intensity in ~35 ps (Fig. 3D). However, the rise in PSII/APC_680_-related fluorescence takes ~40 ps in the dark state and ~55 ps in the light state. Due to the APC_680_ fluorescence contribution at 685 nm, the effect of PBS uncoupling, as discussed in the previous paragraph, is also observed at 685 nm, namely an overall lengthening of the fluorescence decay.

To check whether the observed delay in the rise of fluorescence intensity from ~40 to ~55 ps in the PSII/APC_680_ emission band is due to PBS or to PSII, we also performed streak-camera measurements using 400-nm excitation (Fig S3). At this excitation wavelength, mainly the PSs are excited. In this case the fluorescence decay traces do not differ between the dark and light states, leading to the conclusion that all the observed changes in fluorescence rise and decay kinetics upon 580 nm excitation must be PBS-related. Also the decay traces at the PSI emission maximum (720 nm) of dark and light states upon 400 nm excitation do not show any differences (Fig S3 D). In addition, our results obtained upon 400 nm excitation show that spillover does not play a role in dark-light transitions. The spillover model assumes that the photosystems reorganize in such a way that EET between PSII and PSI changes, which would affect the PSI and PSII fluorescence decay kinetics. However, no difference in the fluorescence decay traces of PSII and PSI was observed between the two states upon 400 nm excitation (Fig S3), showing that spillover does not play a role in dark-light transitions.

These data collectively show that EET from PBS towards PSI occurs equally fast for both the light and dark states (~35 ps) (Fig. 3), while the initial part of the 685-nm fluorescence trace, reflecting EET from PBS to PSII, is clearly slower in the light state (dark: ~40 ps; light ~55 ps). Interestingly, the fact that the rate of EET from PBS to PSII is not faster than to PSI differs from recent *in vitro* results on the PBS-PSII-PSI megacomplex^31^, which showed that EET from PBS towards PSI is slower than towards PSII. Apparently, PBS-PSII-PSI megacomplexes as described in^31^ do not dominate the fluorescence kinetics *in vivo* neither in the dark nor in the light. Either they are not present in large quantities or their properties are different *in vivo* and *in vitro*.

### Global analysis of streak-camera data

**Figure 4:**
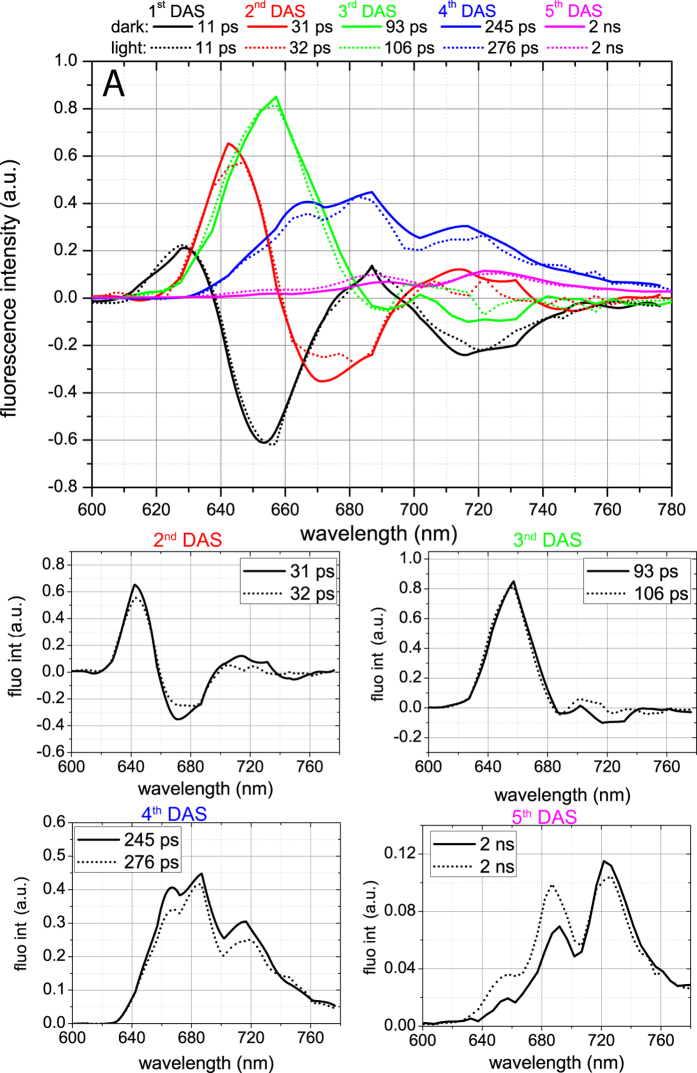
DAS for dark (solid line) and light (dotted line) states obtained from global analysis of streak-camera data measured at 77 K upon 580 nm excitation. (**A**)—The lifetimes of the corresponding DAS are presented in the legend with the corresponding colours. The slowest component was always fixed to 2 ns. This lifetime was obtained independently for many datasets, but due to the limited time window of our setup (800 ps), it was not possible to resolve it in a reliable way. The shape of both fluorescence spectra at t = 0 were identical and they were normalized to each other. (**B**–**E**)— Comparison of individual DAS as presented in (**A**). The first DAS (11 ps) was identical for both measurements and is not presented here. T = 0 and time-integrated spectra are presented in Supporting Material (Fig S4).

In order to study PBS mobility between PSs, we performed global analysis of the streak-camera data obtained upon 580 nm excitation at 77 K. The resulting decay-associated spectra (DAS) are presented in Fig. 4A. Five components were sufficient to describe the data for both the dark and the light state. The 1^st^ DAS (11 ps) has a positive and a negative peak at 630 and 650 nm, respectively, representing EET in the PC rods together with a positive and a negative peak at 690 nm and 720 nm respectively, representing EET within PSI^32^,^38^. The 2^nd^ DAS carries the typical signature of EET from PC towards APC_660_ with a positive peak at 640 nm and a negative one at ~665 nm^32^,^38^. For dark-state cells it also has a positive and a negative band in the 700–760 nm region, representing PSI fluorescence kinetics, which is lacking for light-state cells. The 3^rd^ component represents EET from APC_660_ to APC_680_/Chls with a 100 ± 10 ps time constant as well as APC_660_ decay. The 4^th^ component, decaying with a ~250 ps in the dark and ~275 ps in the light, reflects the sum of several decay processes with peaks at 665 nm, 682 nm, and 715 nm. The 665-nm peak is characteristic for PBSs^31^. The 682-nm peak of the 4^th^ DAS was previously assigned to PSII, while the 715 nm peak is characteristic for PSI^26^. The 5^th^ DAS (2 ns) represents the decay of both PSII at ~695 nm and PSI at ~725 nm, while a small contribution from PC and APC_660_ results in the 650–660 nm band. The 686-nm maximum of the 5^th^ DAS should be dominated by APC_680_, since no PSII-related components with ~1–2 ns lifetime and 686-nm peak position have been resolved before, neither *in vivo* nor *in vitro*^26^,^31^. The 360 ps and 1.3 ns lifetimes have previously been reported for APC_680_ *in vivo* at RT^28^, while at 77 K APC_680_ fluorescence decays with a 2 ns lifetime *in vitro*; thus APC_680_ emission contributes to the 5^th^ DAS and hardly to the 4^th^ DAS in the 685 nm region (Fig. 3).

The individual DAS for the dark and light states were then compared, as presented in Fig 4B–E. The 1^st^ DAS (Fig. 4A) does not differ for the dark and the light state. The 2^nd^ DAS (Fig. 4B) shows higher amplitudes for the positive 640-nm and negative 665-nm peaks in the dark state than in the light state. In the dark state, it also carries a positive and a negative band in the PSI-related region (700–760 nm) that is absent in the light state. The first difference might be caused by a lower number of pigments participating in EET from PC towards APC_660_ in PBSs in the light. In such a case one would expect the energy transfer rate from PBS rods to PBS cores to be faster^39^. However, the rising parts of the APC-associated traces detected at 665 nm and 678 nm (Fig. 3, inserts) do not differ between the dark and light states. Consequently, the decrease in amplitude of the positive 640-nm and negative 665-nm peaks in combination with the disappearance of the positive and negative PSI-related bands in the light state most probably indicate that less energy is transferred from PC and/or APC_660_ to PSI.

Further, also in the 3^rd^ DAS (Fig. 4C), the intensity of the negative PSI-related band is lower in the light state than in the dark state, again indicating that less energy is transferred from PBSs to PSI. In the light state, this DAS shows EET from APC_660_ towards APC_680_ pigments of PBSs and Chls of PSII, both emitting at ~685 nm. However, the amplitude of the negative APC_680_/PSII-related 680–685-nm band is not higher in the light state than in the dark state, suggesting that (most of the) PBSs that energetically uncouple from PSI in the light, do not migrate to PSII. In the light state, a 15 ps longer lifetime for the 3^rd^ DAS was observed, reflecting a decrease of the rate of EET from PBSs to PSII, as discussed in the previous section. PBS energetic uncoupling from PSI should not significantly contribute to the changes of the 3^rd^ DAS lifetime, since it was demonstrated that energy transfer rates in isolated cyanobacterial phycobilisomes are nearly identical to those obtained for intact cells^32^,^39^.

In the 4^th^ DAS, the main differences between dark and light states are the lower PBS-related 665-nm fluorescence and PSI-related 715-nm fluorescence in the light state. The PSII-related 685-nm fluorescence does not seem to differ for the two states, again suggesting that PBSs do not attach to PSII in the light. In the 5^th^ DAS, fluorescence increases by 50% in the PC/APC_660_-related region (640–660 nm), and by 40% in the APC_680_/CP47-related region (685–695 nm), and again PSI emission decreases slightly. The higher long-lived PBS-related fluorescence of the 5^th^ DAS for the light state indicates PBS energetic uncoupling (an increase of the long-time (~ns) DAS amplitudes points at inefficient EET), and the decrease of PSI-related emission in the 4^th^ and 5^th^ DAS shows that the PBSs uncouple from PSI (Fig. 4E).

From global analysis of streak-camera data of the cells in the light and dark states, as presented above, we conclude that a fraction of the PBS population is uncoupled from PSI in the light state. It appears that this uncoupling is not generally followed by antenna attachment to PSII, as can be judged from the 3^rd^–5^th^ DAS.

### Gaussian decomposition of the 4^th^ and 5^th^ DAS

**Figure 5:**
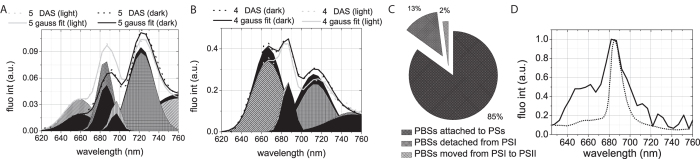
Detailed analysis of the 4^th^ and 5^th^ DAS. Gaussian decomposition of 4^th^ (**A**) and 5^th^ (**B**) DAS obtained from global analysis of time-resolved data in dark (black lines) and light (grey lines) states. The solid lines represent the original DAS, while the dotted lines show the spectrum fitted with the Gaussians. Dark and light grey shaded areas represent (dark and light) Gaussian bands. The corresponding parameters can be found in Table S1. (**C**)— Pie graph representing changes in PBS-PS association during dark-light transition. 85% of the PBSs do not change their associations with the PSs during this transition and remain attached to the photosystems; 15% uncouples from PSI, while only 2% moves from PSI to PSII. (**D**)— Difference spectrum (solid line) obtained by subtracting the 5^th^ DAS for the dark state from that for the light state. Before subtraction, both DAS were normalized to their 722-nm bands. The difference spectrum is compared with the spectrum of isolated PBSs measured at 77 K (dotted line, from (15), red-shifted 5 nm because a different medium was used).

Since the 680–690 nm region of the 4^th^ and 5^th^ DAS is contributed to by APC_680_, PSII, CP47 in PSII, which are spectrally all very close, it is difficult to separate their individual contributions directly by visual inspection. In order to answer the question whether more PBSs are attached to PSII in the light than in the dark state, we performed a Gaussian decomposition of the 4^th^ (Fig. 5A) and 5^th^ DAS (Fig. 5B). Four Gaussians were needed to decompose the 4^th^ DAS for both states, while five Gaussians were needed for the 5^th^ DAS (Fig. 5A,B, Table S1).

First, the amplitude differences for the PSII-related Gaussians of the dark and light states were checked. The area of the PSII-related Gaussian of the 4^th^ DAS (maximum at 685 nm) is the same for the dark and the light state. If more PBSs would attach to PSII in the light, this area would be expected to be higher. However, the area of the CP47-related Gaussian in the 5^th^ DAS, centred at 695-nm, is higher in the light state by 25%. This increase suggests that PBSs attach to PSII only at the side of the red CP47 Chls. This is in agreement with earlier claims by Bittersmann and Vermaas^40^, who suggested the involvement of CP47 in EET between PBS and PSII. In that study, EET from PBS towards PSII in several PSII mutants of Synechocystis was measured by time-resolved fluorescence spectroscopy, and it was found that, in the absence of CP47, hardly any excitation energy is transferred to PSII.

Successively, the differences of the PBS- and PSI-related Gaussians for the two states were analysed. The area of the 665-nm PBS-related Gaussian in the 4^th^ DAS is 13% smaller in the light than in darkness. This is almost identical to the total decrease in dark-to-light transition of two PSI-related Gaussians in the 4^th^ and 5^th^ DAS (12%). In the 5^th^ DAS, the areas of two PBS-related Gaussians, centred at 656 and 685 nm, increase substantially in the light, while the increase of the 695-nm Gaussian area constitutes only ~2% of the PBS-related Gaussian area in the 4^th^ DAS. We conclude that around 15% of the PBSs that energetically uncouple from PSI in the light (13% of the total PBS pool) attach to PSII at the CP47 side, while 85% of them remain uncoupled (Fig. 5C).

Due to the fact that the increase of the area of the PC-related emission band is 50% higher than that of the APC_680_-related Gaussian in the light compared to darkness, the uncoupled PBSs should contain more PC than ‘conventional’ PBSs^27^. In line with this, the 2^nd^ DAS shows that fewer PC pigments transfer energy to PSI in the light than in the dark state, while there is no change in the total amount of APC_660_ pigments transferring their excitation energy to APC_680_ and to the PSs.

The steady-state fluorescence spectrum of the uncoupled PBS pool was visualized by plotting the intensity difference for the light and dark states in the 5^th^ DAS, after the spectra were normalized to the PSI emission (Fig. 5D). The ratio of the 650–670/685 nm emission bands of the difference spectrum, namely 0.5:1.0, differs substantially from the reported ratio for isolated PBSs, namely 0.15:1.0 27. The fact that the 1^st^, 2^nd^ and 3^rd^ DAS, which represent EET within the PBSs, almost do not change upon the dark-light shift, implies that the enhanced emission in the 650–670 nm region is not due to disassembled PBSs. If this were not the case, variations in the lifetimes and the amplitudes of the DAS would be observed, as in cyanobacterial mutants with truncated PBSs^39^. Kondo *et al.* recently reported the existence of ‘alternative’ PBSs, namely CpcG2-PBSs, consisting of only PC rods with a CpcG2 linker that seem to preferentially transfer energy to PSI^41^, while Watanabe *et al.* demonstrated that this PBS type emits fluorescence at 665 nm at 77 K^42^. Therefore, the enhanced emission (as compared to conventional hemi-discoidal CpcG1-PBS) in the 665-nm region of the difference spectrum in Fig 5D indicates that the uncoupled PBS pool contains two types of PBSs: hemi-discoidal CpcG1-PBS and CpcG2-PBS, missing the APC core. In^36^ it was concluded that the latter PBS type is not involved in state transitions, but we demonstrate its involvement through uncoupling from PSI in the light state during dark-to-light transition. This uncoupling in the light might also explain why in cells without CpcG2, where only ‘conventional’ hemi-discoidal PBSs are present, the extent of state transitions, observed by 77 K fluorescence, is lower than in wild-type cells. The reason for PBSs to uncouple in the light is not clear and requires further investigation. A larger PSI antenna during the dark-to-light transition might be beneficial to increase the rate of plastoquinone re-oxidation by PSI. In this way PSII could be provided with an adequate amount of electron acceptors to re-reduce. Once sufficient reducing power is achieved, a fraction of the PBSs might uncouple from PSI to avoid over-oxidation of the electron transport chain.

Finally, it is worth mentioning that the observed dark-light changes may be related to changes in the width of the inter-thylakoid space^43^,^44^,^45^. It has been demonstrated that in Synechocystis cells the distance between thylakoid membrane pairs increases in the light^43^. In this way, distance variation between thylakoid membrane pairs might regulate (CpcG2)-PBS attachment to the photosystems.

In conclusion, our study demonstrates that the rearrangement of the photosynthetic apparatus during dark-light transitions does not involve the photosystems, i.e. there is no spillover, but mainly involves reversible phycobilisome decoupling from PSI. Around 13% of the total pool of phycobilisomes uncouples from PSI in the light, while only 2% reattaches to PSII at the CP47 side. Finally, it is concluded that the PBS-PSII-PSI megacomplexes as we know them from recent *in vitro* work are not prominently active or needed *in vivo* in growth light conditions.

## Materials and Methods

### Strains and Growth Conditions

*Synechocystis* sp. PCC 6803 cells were grown at 30 °C in BG-11^46^ buffered with 20 mM HEPES-NaOH (pH 7.5) and sodium carbonate was omitted from the culture ingredients. White light was used for illumination at 50 μmol photons m^−2^ s^−1^. The cultures were grown in 100-ml flasks with a culture volume of 20 ml. The flasks were shaken at 100 rpm. Fig S5 shows the profile of the light that was used for cultivation.

### Sample preparation

*Synechocystis* sp. PCC 6803 cells were harvested during the logarithmic phase (optical density at 750 nm (OD_750_) between 0.6 and 1.1 for a pathlength of 1 cm), resuspended in fresh BG-11 medium, and adjusted to OD_670 _= 0.2–0.3 (again for 1 cm), using a spectrophotometer with integrating sphere. The cells were either taken directly from the growth chamber (light state) or after keeping them in the dark for 10 min (dark state). For the fluorescence measurements samples in both states were collected in glass Pasteur pipettes with ~1 mm diameter to avoid reabsorption effects and then frozen at 77 K by immediate immersion in liquid nitrogen. The reversibility of the light-to-dark transition was checked by recording the 77 K steady-state fluorescence spectrum of light-adapted cells (10 min light adaptation, Fig S1, light 2) after a 10 min dark-adaptation period. The obtained spectrum was identical to the spectrum of the cells in the light state.

### Steady-State Fluorescence

Steady-state fluorescence spectra were recorded with a Fluorolog FL3–22 spectrofluorimeter (Horiba Jobin Yvon, Edison, NJ) and corrected for wavelength-dependent sensitivity of the detection and fluctuations in lamp output. The excitation wavelength was 580 nm; a band-pass of 2 nm was used for both the excitation and emission monochromator. Instead of the commonly used normalization to the emission peaks, Rhodamine B was used as an internal standard to independently check the fluorescence emission changes in the whole range of steady-state spectra. The standard was added to the culture just before freezing in liquid nitrogen. Fluorescence emission spectra were recorded using an integration time of 0.2 s. Each measurement was repeated three times and the results were averaged. Steady-state emission spectra in the presence and absence of Rhodamine B do not show significant differences above 650 nm.

### Time-resolved fluorescence measurements

Time-resolved emission spectra were recorded using a synchroscan streak-camera system as described in^47^. An excitation wavelength of 580 nm was used to excite preferentially the PBSs, while 400 nm was used to preferentially excite Chl *a* in the PSs. The laser power was 60 μW, the time-window 800 ps, the spot size 100 μm, and the repetition rate 250 kHz. An average of 100 images, all measured for 10 s, was used to achieve a high signal/noise ratio. Samples did not change during the measurement as was confirmed by comparing fluorescence decay traces in the beginning and in the end of the measurement series (Fig S6). Before analysis, the images were corrected for background signal and detector sensitivity and sliced up into traces of 5 nm. The streak-camera images were analysed as described previously^48^. In short, a number of parallel, non-interacting kinetic components was used as a kinetic model, so the total dataset was fitted with function *f (t, λ):*


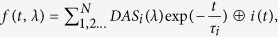


where *DAS*_*i*_ (Decay Associated Spectra) is the amplitude factor associated with a decay component *i* having a decay lifetime *τ*_*i*_. A Gaussian-shaped instrument response function 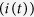 was used as input for the analysis with the width as a free fitting parameter. FWHM values obtained from the fitting procedure were in the range of 12 ± 1 ps.

## Additional Information

**How to cite this article**: Chukhutsina, V. *et al.* Cyanobacterial Light-Harvesting Phycobilisomes Uncouple From Photosystem I During Dark-To-Light Transitions. *Sci. Rep.* **5**, 14193; doi: 10.1038/srep14193 (2015).

## Supplementary Material

Supplementary Information
