# Supplementary material for: Cyanobacterial Light-Harvesting Phycobilisomes Uncouple From Photosystem I During Dark-To-Light Transitions
Source: Sci Rep. 2015 Sep 21;5:14193. doi: 10.1038/srep14193 (PMC4585685; doi:10.1038/srep14193)
Supplement: Supplementary Information [file srep14193-s1.pdf]

Cyanobacterial Light-Harvesting Phycobilisomes Uncouple From Photosystem I During Dark-To-Light Transitions

Volha Chukhutsina, Luca Bersanini, Eva-Mari Aro and Herbert van Amerongen*

SUPPORTING INFORMATION

1. Reversibility of dark-to-light transitions

We checked the reversibility of dark-light and light-dark transitions by recording 77 K steady-state fluorescence spectrum on the cells that were adapted back for 10 min to light after reaching the dark state (10 min of dark-adaptation) (Fig. S1, light 2). The obtained spectrum was similar to that of the light state.

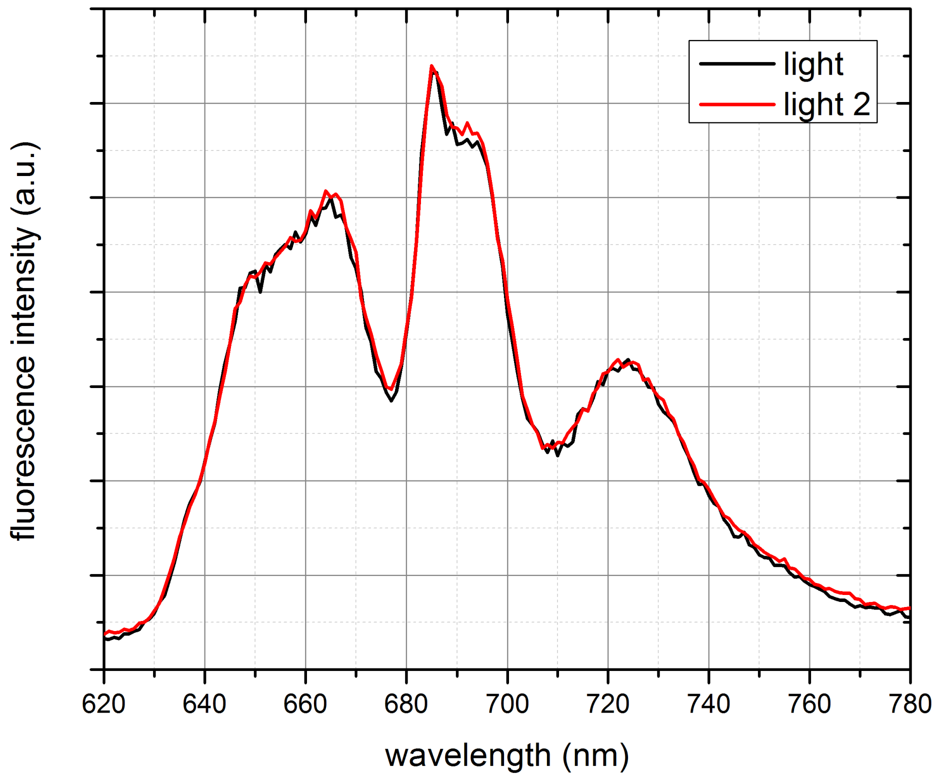

Fig. S1. Comparison of steady-state fluorescence spectra in light and light 2 (10 min of light-adaptation after dark state) states measured at 77K upon 580 nm excitation.

2. Gaussian decomposition of 4th and 5th DAS

The 4th DAS in both states were described by four Gaussians centered at 666 nm, 686 nm, 713-714 nm and 750 nm, respectively (Fig. 6, Table S1). The 666/667-nm Gaussians represent emission from PC and APC pigments in PBSs, while the 686-nm band should be assigned to PSII and maybe (partly) to APC_{680}. The last two Gaussians should be mostly attributed to PSI, while some small contributions from PSII and PBS vibrational bands are also present. The steady-state fluorescence spectrum of PSII is well known and has two fluorescence emission peaks (685 nm and 695 nm) of around equal amplitudes, arising from CP43 as well as CP47 and from low-energy Chls in CP47, respectively (1). Both *in vivo* and *in vitro* time-resolved studies demonstrated that Chls emitting at around 685 nm in PSII contribute to the fastest part of PSII fluorescence decay (200-400 ps lifetime) at 77K, while red-absorbing Chls in CP47 decay with ~1 ns lifetime (2, 3). The 5th DAS were decomposed with five Gaussians centered at 656 nm, 686 nm, 696 nm, 722 nm and 760 nm, respectively. Again the two most red-shifted Gaussians should be mostly attributed to PSI. The 656-nm Gaussian mostly represents emission from PC rather than from APC because of its peak position. The 696-nm Gaussian is mainly due to CP47 emission, although the emission from APC_{680} of the PBSs in this region cannot be completely ignored. The 686-nm Gaussian of the 5th DAS, due to its lifetime, should be mostly due to APC_{680} (2). Because its peak position is quite close to that of the 1 ns component , previously assigned to radical pair formation in PSII, some PSII contribution cannot be completely excluded (4).

Table S1. Decomposition of 4th and 5th DAS into Gaussians for dark and states.

	Gauss 1	Gauss 2	Gauss 3	Gauss 4	Gauss 5
			WT_{Dark}		
4th DAS					
Ampl *100, a.u.	41	23		28	8
Peak, nm	666	687		713	751
FWHM, nm	19.5	8.5		22	34
5th DAS					
Ampl *100, a.u.	1.9	5.2	2.0	9.4	4.2
Peak, nm	656	686	696	722	760
FWHM, nm	21	11	7.5	18	40
			WT_{Light}		
4th DAS					
Ampl *100, a.u.	34	24		23	9
Peak, nm	667	686		714	750
FWHM, nm	21	8.5		21	30
5th DAS					
Ampl *100, a.u.	3.6	7.8	3.4	8.9	3.7
Peak, nm	656	685	696	722	760
FWHM, nm	21.5	11	7.7	19	40

3. Data reproducibility

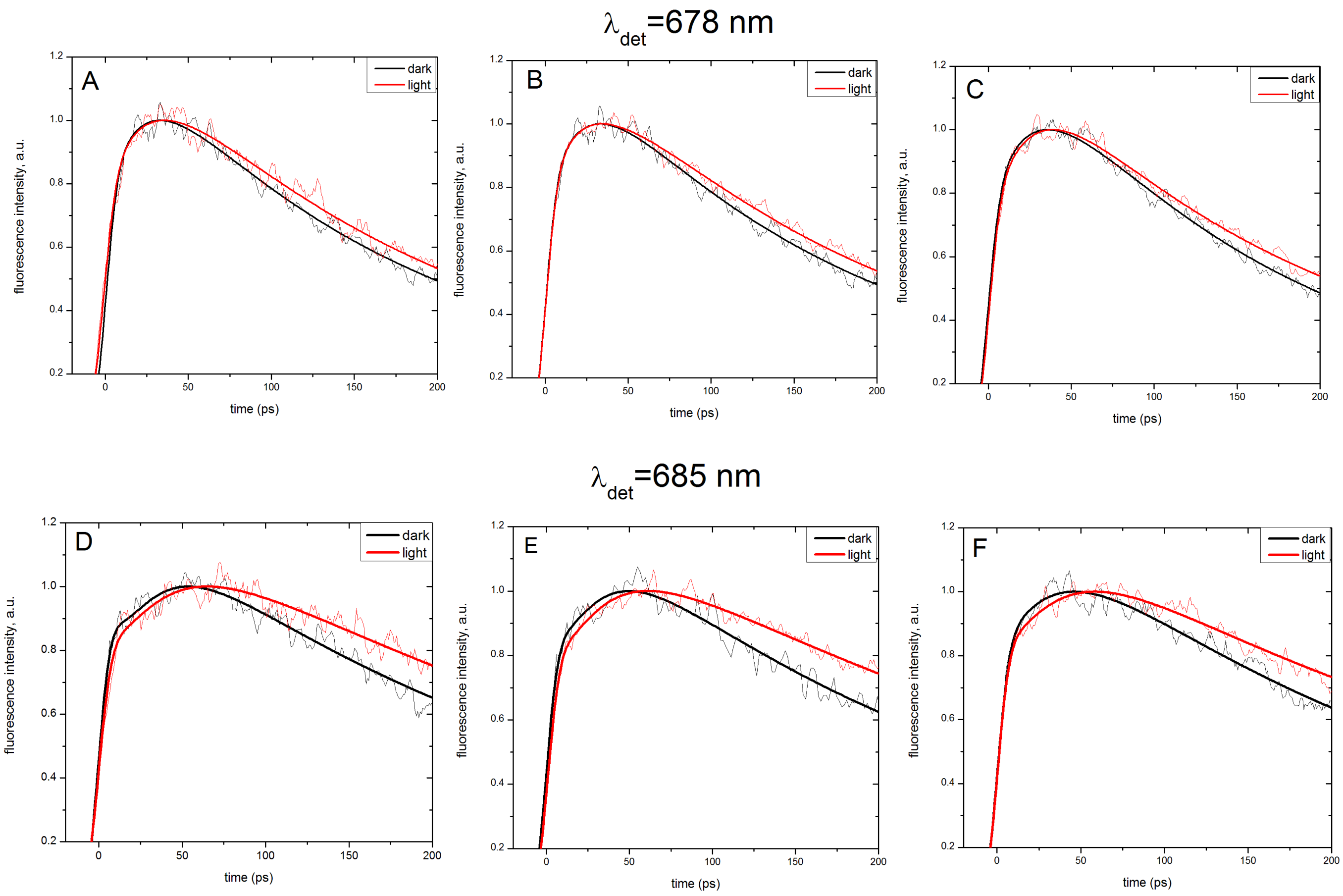

Fig. S2. Fluorescence kinetics of *Synechocystis sp. PCC 6803* cells excited at 580 nm and detected at 678 nm (A, B, C) and 685 nm (D, E, F) measured for three independent generations.

Each experiment was repeated at least 3 times for different generations of cells. The streak-camera images as well as fluorescence decay curves for every measurement appeared to be very similar in case of independent generations and the difference between 'light' and 'dark' states were consistent. Fig. S2 shows the initial part of fluorescence kinetics in light and dark measured on different generations of the cells detected at 678 nm and at 685 nm. As can be seen the differences between EET rates for light and dark states are always present at 685 nm and are not observable at 678 nm.

4. Time-resolved measurements upon 400 nm excitation

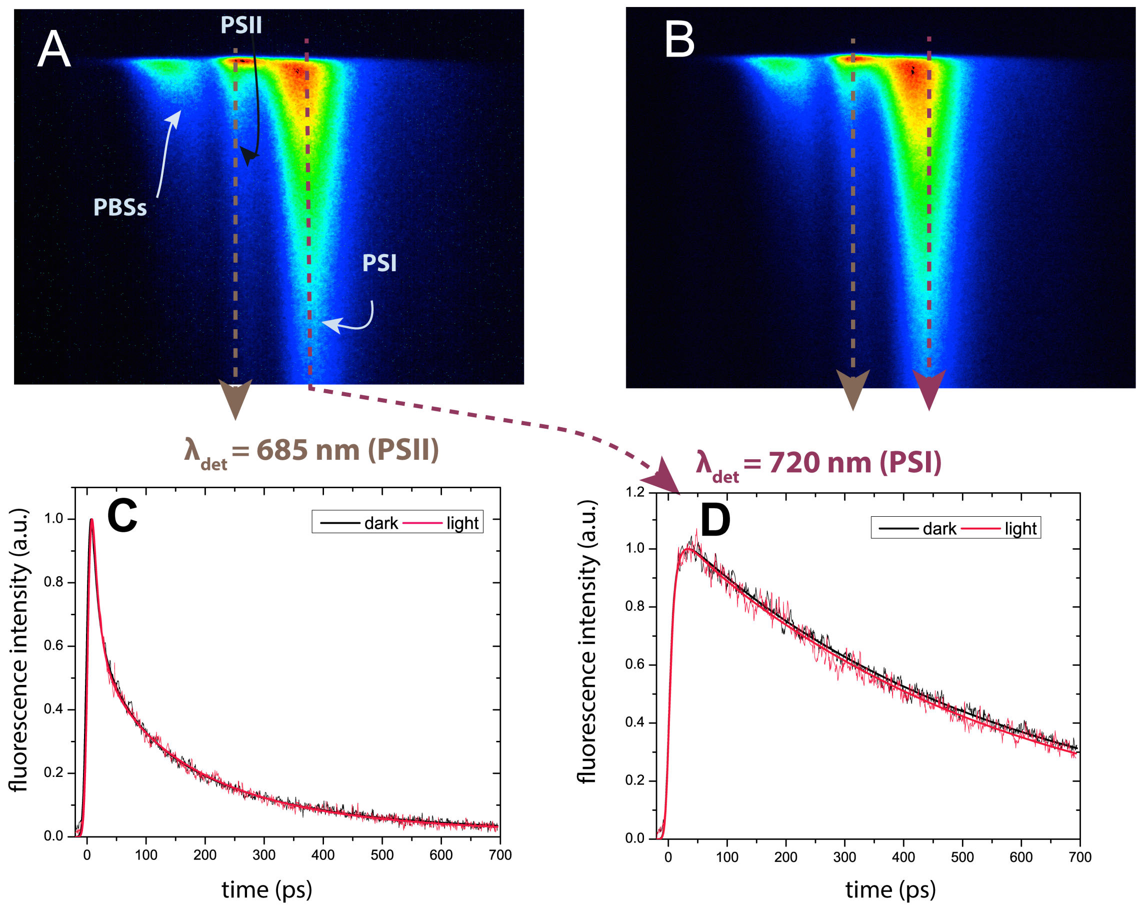

Fig. S3. Time-resolved fluorescence of *Synechocystis sp. PCC 6803* measured in dark- (A) and light-adapted states (B) at 77K upon 400 nm excitation. (A, B) Streak-camera images. False colors indicate the fluorescence intensity. (C, D) Representative decay traces were taken at PSII (C) and PSI (D) emission maxima: 685 nm and 720 nm, respectively. For better comparability, the traces in light (red lines) and dark (black lines) states were normalized to their maxima.

4. T=0 and time-integrated spectra

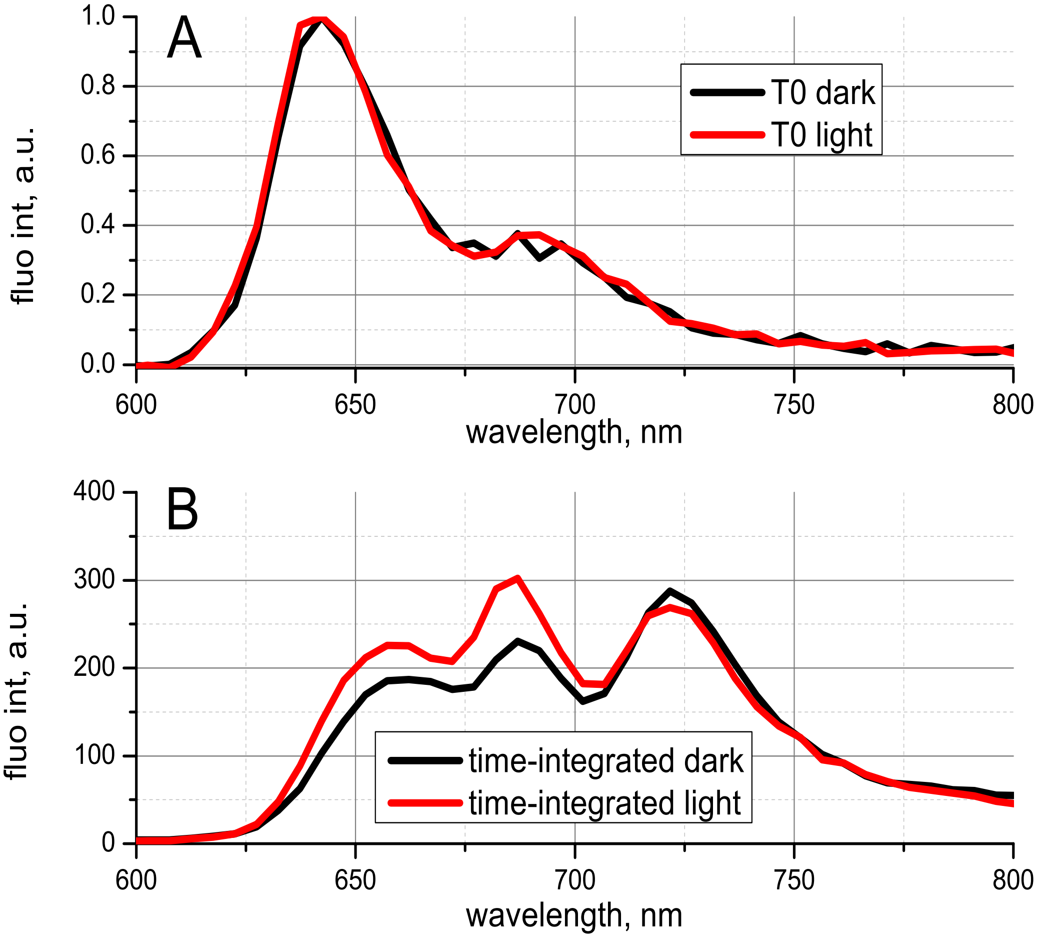

Fig. S4. T=0 (A) and time-integrated spectra (B) obtained from DAS presented in Fig 4.

The total spectra at t=0 are equal to the sum of the various corresponding DAS for *Synechocystis sp. PCC 6803* measured in a particular state: dark- or light- adapted. All DAS obtained from global analysis of datasets for a particular state were summed and normalized in the maximum. The resulting spectra are independent of the state of the cells (Fig. S4 A). Time-integrated spectra were obtained by summing up all decay component yields (DAS amplitude times lifetime) in a particular state. Relatively higher contribution of the 722-nm band as compared to the one resolved from steady-state spectra (Fig. 1) is due to the fact that ≳1 ns components cannot be accurately resolved from streak-camera measurements due to the limited time-detection window (800 ps).

5. Spectral profile of the growth light

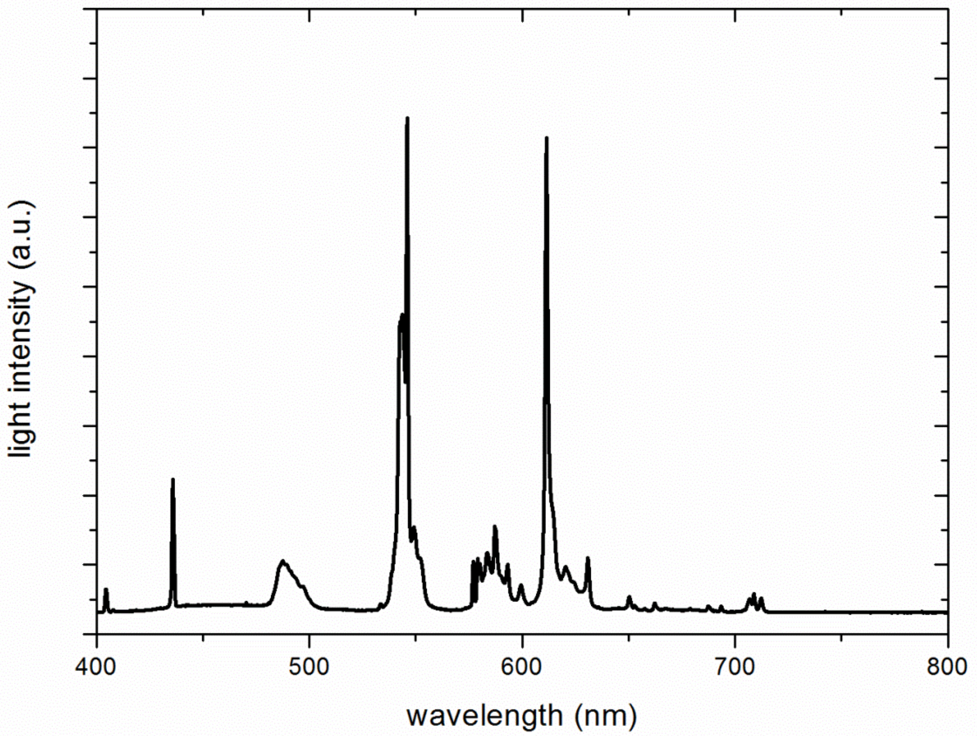

Fig. S5. Spectral profile of the light used for cultivation.

5. Comparison of fluorescence kinetics in the beginning and in the end of measuring series

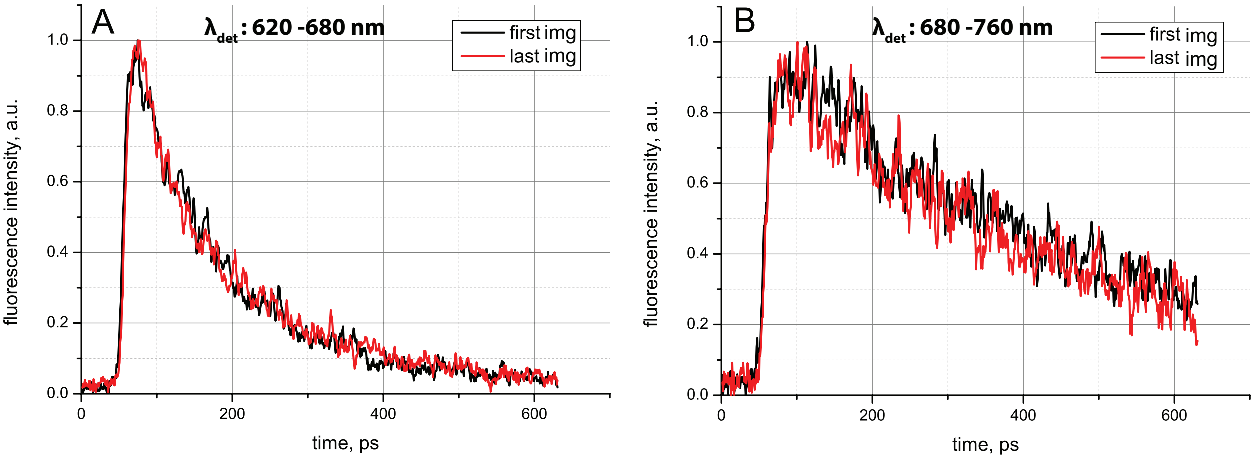

Fig. S6. Comparison of fluorescence kinetic traces from the first and last collected image of a measurement series (100 images in total) of *Synechocystis sp. PCC 6803* (light state). Decay traces were summed within two detection regions: 620-680 nm (A) and 680-720 nm (B).

To confirm that the samples do not change during measurements, we compared fluorescence decay kinetics of the first and last image of a measurements series. In Fig S5, summed decay traces of *Synechocystis sp. PCC 6803* measured in the light state are presented. The fluorescence kinetics does not changes during the measurements neither in the 620-680 nm (Fig S5 A) nor in the 680-720 nm (Fig S5 B) region.

1. Andrizhiyevskaya EG*, et al.* (2005) Origin of the F685 and F695 fluorescence in photosystem II. *Photosynthesis research* 84(1-3):173-180.
2. Liu HJ*, et al.* (2013) Phycobilisomes Supply Excitations to Both Photosystems in a Megacomplex in Cyanobacteria. *Science* 342(6162):1104-1107.
3. Tian L, Farooq S, van Amerongen H (2013) Probing the picosecond kinetics of the photosystem II core complex in vivo. *Phys Chem Chem Phys* 15(9):3146-3154.
4. van der Weij-de Wit CD, Dekker JP, van Grondelle R, van Stokkum IH (2011) Charge separation is virtually irreversible in photosystem II core complexes with oxidized primary quinone acceptor. *The journal of physical chemistry. A* 115(16):3947-3956.
